# Prevalence and Trends in Gestational Diabetes Mellitus Among Women in the United States, 2006–2017: A Population-Based Study

**DOI:** 10.3389/fendo.2022.868094

**Published:** 2022-06-06

**Authors:** Tao Zhou, Shan Du, Dianjianyi Sun, Xiang Li, Yoriko Heianza, Gang Hu, Litao Sun, Xiaofang Pei, Xiaoyun Shang, Lu Qi

**Affiliations:** ^1^ School of Public Health (Shenzhen), Shenzhen Campus of Sun Yat-sen University, Shenzhen, China; ^2^ Department of Epidemiology, School of Public Health and Tropical Medicine, Tulane University, New Orleans, LA, United States; ^3^ Department of Epidemiology and Biostatistics, School of Public Health, Peking University Health Science Center, Beijing, China; ^4^ Chronic Disease Epidemiology Laboratory, Pennington Biomedical Research Center, Baton Rouge, LA, United States; ^5^ Department of Public Health Laboratory Sciences, West China School of Public Health, Sichuan University, Chengdu, China; ^6^ Department of Pediatrics, Children’s Hospital New Orleans, New Orleans, LA, United States; ^7^ Department of Nutrition, Harvard T.H. Chan School of Public Health, Boston, MA, United States; ^8^ Channing Division of Network Medicine, Department of Medicine, Brigham and Women’s Hospital and Harvard Medical School, Boston, MA, United States

**Keywords:** gestational diabetes, trend, prevalence, risk factors, National Health Interview Survey

## Abstract

The prevalence of gestational diabetes mellitus (GDM) has increased with the increasing rate of obesity. However, national data on the prevalence and secular trends of GDM during the past decade in the United States are lacking. This study included 37,357 women aged more than 18 years and who had ever been pregnant from the National Health Interview Survey (NHIS). We examined GDM prevalence in 2006, 2016, and 2017, with age-standardized to the US population in 2000. We found that the prevalence of GDM per 100 people increased from 4.6 (95% CI, 4.1–5.1) in 2006 to 8.2 (95% CI, 7.5–8.9) in 2016 (test for difference; P <0.001), with a relatively increased rate of 78%. Non-Hispanic white women tended to have a lower increase (2.8%) than non-Hispanic black women (3.8%), Hispanic women (4.1%), and women of other race/ethnicity (8.4%). The prevalence of GDM in non-Hispanic white women was higher than that in non-Hispanic black women in 2006 (4.8% vs 3.5%, P = 0.006); such differences became non-significant in 2016 (P = 0.72). Additionally, the increase of GDM from 2006 to 2016 tended to be more evident among women who were overweight (25≤ BMI ≤30 kg/m^2^), physically inactive, and with family income below the poverty threshold than women in other BMI ranges, with more physical activity, and with higher incomes. The prevalence of GDM per 100 people in 2017 was 8.4 (7.6–9.2), and there was no significant change in the overall and subgroup prevalence compared with 2016. Collectively, in the United States, the prevalence of GDM continuously increased, nearly doubled, from 2006 to 2016, and then leveled off in 2017. The increase appeared more marked among the minority populations and subpopulations with overweight people, insufficient activity, and family incomes below the poverty threshold.

## Introduction

Gestational diabetes mellitus (GDM) is a condition in women with impaired glucose tolerance with the onset or first recognition during pregnancy (1). In recent years, GDM has become an increasing public health concern due to its adverse implications for maternal and child health (2–6). In the short-term, GDM increases adverse pregnancy outcomes (2); and in the long-term, GDM carries an increased risk of developing type 2 diabetes for the mothers and an elevated risk of various cardiometabolic disorders in the offspring (7–9).

The prevalence of GDM has increased during recent decades in the United States. In a study using the National Hospital Discharge Survey database, the estimated prevalence of GDM in the United States was 5.8% in 2008–2010, with an absolute increase of 5.5% and a relative increase of 23 folds since 1979–1980 (10). More specifically, from 1989–1990 to 2003–2004, the prevalence of GDM increased from 1.9 to 4.2%, with a relative increase of 122% (11). This increasing trend was also observed in regional data (12–15). However, inconsistent data were also reported; for example, no significant change in GDM prevalence from 2007 (8.1%) to 2010 (8.5%) was observed in the Pregnancy Risk Assessment Monitoring System (PRAMS) including 21 states of the United States (5). Additionally, national data on the most recent prevalence and secular trend of GDM prevalence in the United States are lacking. Moreover, little is known about whether GDM prevalence and changing trends differ with race/ethnicity and other population characteristics.

The National Health Interview Survey (NHIS) is a national cross-sectional survey that collects health and lifestyle information from sample participants representing the U.S. population. This study aimed to determine the temporal trend of GDM prevalence among pregnant women from 2006 to 2016, and 2017 using data from NHIS. We particularly analyzed the secular trend of GDM prevalence and compared the differences in subgroups categorized by race/ethnicity, Body Mass Index (BMI), physical activity, and socioeconomic status.

## Materials and Methods

### Study Design and Participants

The NHIS is an ongoing national cross-sectional survey that monitors the health of the U.S. population. Using a stratified, multistage sampling design, NHIS conducts personal household interviews to collect health and lifestyle information from sample participants who represent the U.S. population. One adult was randomly selected from each household to answer the questionnaire. The annual response rate of NHIS was nearly 90% of the eligible households in the sample.

### GDM Assessment

We examined GDM prevalence in 2006, 2016, and 2017. A total of 37, 357 women aged more than 18 years and who had ever been pregnant were included in the current study. In 2006, GDM was asked among 13,525 women aged more than 18 years and who had ever been pregnant, in response to the question “Before you were told you had diabetes, were you ever told that you had diabetes or gestational diabetes while you were pregnant” (cases with diabetes, N = 138) and “Have you ever been told that you had diabetes or gestational diabetes while you were pregnant?” (cases without diabetes, N = 430). In 2016 and 2017, GDM prevalence was measured in 13,650 and 11,041 women by responding to the questions: “Were you ever told by a doctor or other health professional that you had diabetes, sugar diabetes, or gestational diabetes during pregnancy?” (N = 974 and 799, respectively). Related variables with values “Never been pregnant”, “Refused to answer”, “Not ascertained” or “Don’t know” were set as missing, leaving 12,728 participants in 2006, 13,612 in 2016, and 11,071 in 2017. Though GDM was self-reported and not been objectively validated in this study, previous studies suggested high validity of self-reported diagnosis of GDM (16).

### Data Assessment

A standardized questionnaire was used to collect information on age, sex, race/ethnicity family income, physical activity, body weight, and height. Stratified analyses were performed to assess the prevalence in subgroups according to different race/ethnicity, BMI categories, physical activity level, and family income.

Race/ethnicity was categorized as Hispanic, non-Hispanic white, non-Hispanic black, and non-Hispanic for all other race/ethnicity groups. BMI was calculated as weight in kilograms divided by height squared. Normal-weight, overweight, and obesity groups were defined by BMI levels (<25 kg/m^2^, 25–30 kg/m^2^, and >30 kg/m^2^, respectively). Based on imputed household income, income was categorized by the ratio of family income to the poverty threshold (<100%, 100–190%, 200–399%, and >400%). Total minutes of physical activity (TPA) was calculated as the sum of the light-moderate PA min and vigorous PA min multiplied by 2. Then, insufficiently active was defined as (TPA) <150 min/wk, sufficiently active as 150≤ TPA ≤300, and highly active as TPA >300 min/wk.

### Statistical Analysis

Characteristics of study participants in 2006 and 2016 were reported in unweighted and sample-weighted mean and standard error (SE) for continuous variables and numbers and percentages for categorical variables. We used χ^2^ tests to test differences in the frequency of stratification factors. The SURVEYREG procedure in SAS was used to test differences between continuous variables and the prevalence of GDM. We first compared the prevalence between 2006 and 2016, and then 2016 and 2017. A Z-test was used to compare the two prevalence estimates. All calculations were weighted to represent the general female adult population aged 18 years or older in the US. We examined GDM prevalence age-standardized to the U.S. population in 2000. The imputation of family income was conducted by CDC using multiple-imputation methodology. For all analyses, weights, strata, and clusters in the NHIS design were taken into account as recommended by the CDC. All statistical analyses were performed with the use of SAS version 9.4 (SAS Institute, Inc., Cary, NC). Two-sided p-values of <0.05 were considered significant.

## Results

**Table 1** shows the characteristics of the participants in 2006 and 2016, respectively. A total of 26,340 women were included in the two years. The mean age of participants was 47.3 ± 0.2 years in 2006 and 51.6 ± 0.2 years in 2016 (P <0.001). The mean BMI increased from 27.1 ± 0.1 kg/cm^2^ in 2006 to 28.1 ± 0.1 kg/cm^2^ in 2016 (P <0.001). The composition of race/ethnicity significantly differed between the two surveys, with more Hispanic and minority populations and less non-Hispanic white in 2016 compared with that in 2006 (P <0.001). Differences in the composition of family income and physical activity were also observed (P <0.001 and P = 0.007, respectively).

**Table 1:** Characteristics of participants in 2006 and 2016.

	2006			2016			*P* ^d^
	No.	Unweighted	Weighted	No.	Unweighted	Weighted	
Age, year	12,728	48.4 ± 18.2^a^	47.3 ± 0.2	13,612	54.7 ± 17.5	51.6 ± 0.2	<0.001
BMI, kg/m^2^	11,881	27.3 ± 6.6	27.1 ± 0.1	12,954	28.0 ± 6.7	28.1 ± 0.1	<0.001
<25	5,148	43.3^b^	45.3 (44.1–46.6)^c^	4,896	37.8	37.8 (36.7–39.0)	
25–30	3,521	29.6	28.6 (27.6–29.5)	3,942	30.4	30.6 (29.5–31.6)	
>30	3,212	27.0	26.1 (25.1–27.1)	4,116	31.8	31.6 (30.5–32.8)	
Race/ethnicity							<0.001
Hispanic	2,183	17.2	12.3 (11.5–13.0)	1,641	12.1	15.6 (14.1–17.1)	
Non-Hispanic white	7,471	58.7	69.9 (68.7–71.0)	9,361	68.8	64.5 (62.7–66.4)	
Non-Hispanic black	2,389	18.8	12.6 (11.8–13.4)	1,774	13.0	13.0 (11.9–14.0)	
Other race/ethnicity	685	5.4	5.3 (4.8–5.8)	836	6.1	6.9 (6.2–7.7)	
Poverty ratio category							<0.001
<100	2,432	19.1	14.1 (13.2–14.9)	2,067	15.5	13.5 (12.6–14.3)	
100–199	2,869	22.5	19.9 (19.0–20.8)	2,845	21.3	19.5 (18.6–20.4)	
200–399	3,753	29.5	30.7 (29.6–31.9)	3,891	29.2	29.3 (28.2–30.4)	
400+	3,674	28.9	35.2 (33.9–36.5)	4,531	34	37.7 (36.3–39.2)	
Physical activity							0.007
Insufficiently active	351	9.7	9.8 (8.7–11.0)	530	10.0	10.3 (9.2–11.4)	
Sufficiently active	812	22.4	22.4 (20.6–24.2)	1,017	19.2	18.8 (17.5–20.2)	
Highly active	2,465	67.9	67.7 (65.8–69.7)	3,760	70.9	70.9 (69.2–72.5)	

a Values are % or mean ± SD.

b Values are %.

c Values are % (95 CI%).

d Comparing weighted variable between 2006 and 2016.

**Figure 1:**
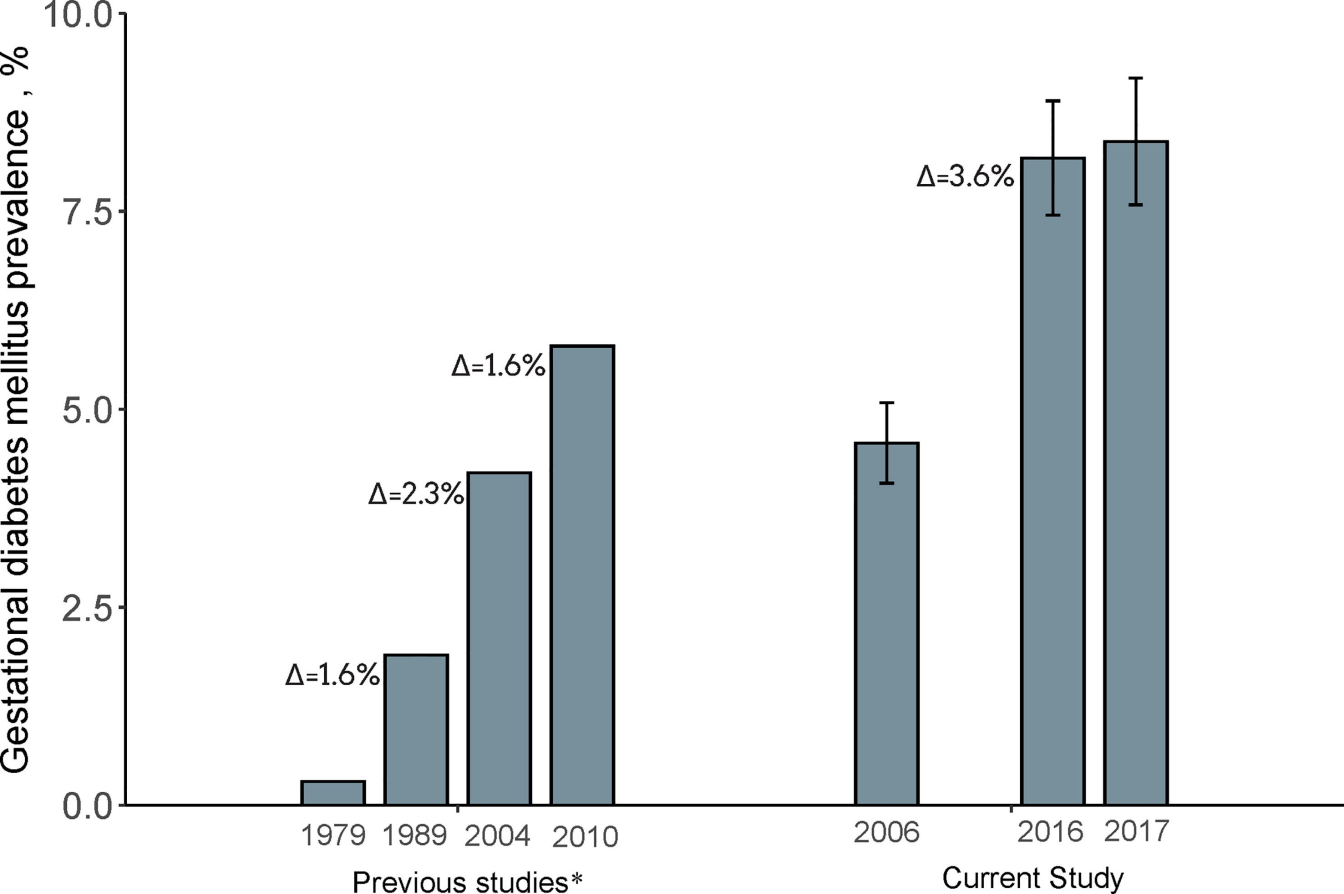
Prevalence of Gestational diabetes mellitus in 2006, 2016, and 2017. Data of the current study were expressed as Estimate ± 95% confidence interval. GDM prevalence was calculated with age-standardized to the U.S. population in 2000. N = 12,728 in 2006, 13,612 in 2016, and 11,071 in 2017. ^a^Date for comparisons were obtained from previous studies with the use of National Hospital Discharge Survey database^1–2^. Δ indicated absolute increase compared with the prevalence of last time period. 1. Getahun et al., *Am J Obstet Gynecol*. 2008. 2. Lavery et al., *BJOG An Int J Obstet Gynaecol*. 2017.

**Figure 2:**
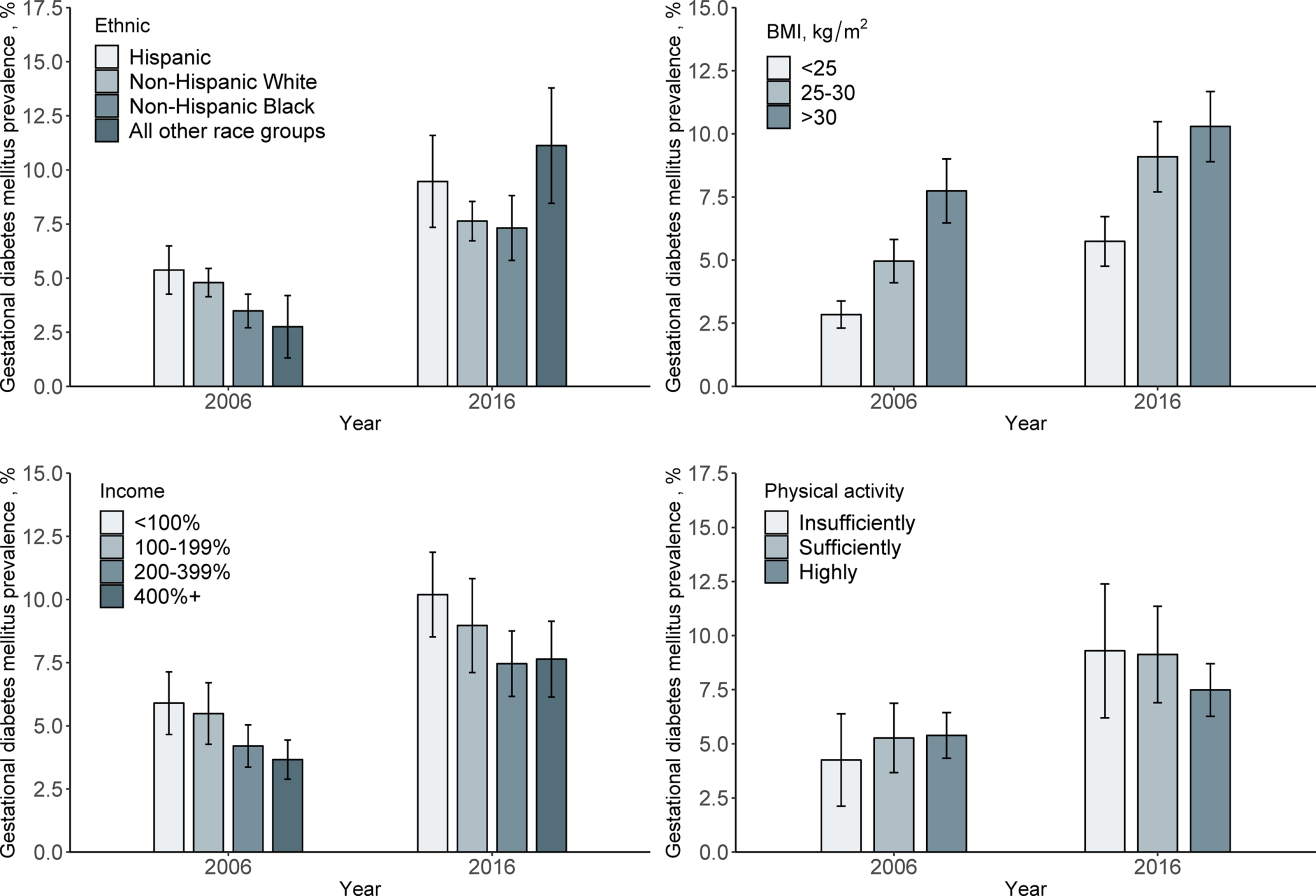
Prevalence of Gestational diabetes mellitus in 2006 and 2016 by demographic variables. Data were expressed as Estimate ± 95% confidence interval. GDM prevalence was calculated with age-standardized to the U.S. population in 2000.

Among the whole study populations, the age-standardized prevalence of GDM per 100 people increased from 4.6 (95% CI, 4.1–5.1) in 2006 to 8.2 (95% CI, 7.5–8.9) in 2016, with an absolute increase of 3.6% and a relative increase of 78% (P <0.001) (**Figure 1**). When populations with various race/ethnicity were compared, non-Hispanic white women showed less increase (2.8%) than non-Hispanic black women (3.8%), Hispanic women (4.1%), and women of other race/ethnicity (8.5%). Notably, the prevalence of GDM in non-Hispanic white women was higher than in non-Hispanic black women in 2006 (P = 0.001). However, such differences became non-significant in 2016 (P = 0.72) (**Table 2** and **Figure 2**).

**Table 2:** Trend in diagnosed Gestational diabetes mellitus of participants in 2006 and 2016^a^.

	2006		2016		Change,% (95% CI)	*P*
	Case No.	% (95% CI)	Case No.	% (95% CI)		
All	568	4.6 (4.1–5.1)	974	8.2 (7.5–8.9)	3.6 (2.7, 4.5)	<0.001
BMI, kg/m^2^						
<25	143	2.8 (2.3–3.4)	241	5.7 (4.8–6.7)	2.9 (1.8, 4.0)	<0.001
25–30	169	5.0 (4.1–5.8)	298	9.1 (7.7–10.5)	4.1 (2.5, 5.8)	<0.001
>30	226	7.7 (6.5–9.0)	385	10.3 (8.9–11.7)	2.6 (0.7, 4.4)	0.008
Race/ethnicity						
Hispanic	144	5.4 (4.3–6.5)	160	9.5 (7.4–11.6)	4.1 (1.7, 6.5)	<0.001
Non-Hispanic white	315	4.8 (4.1–5.4)	583	7.6 (6.7–8.5)	2.8 (1.7, 4.0)	<0.001
Non-Hispanic black	86	3.5 (2.7–4.3)	128	7.3 (5.8–8.8)	3.8 (2.2, 5.5)	<0.001
Other race/ethnicity	23	2.8 (1.3–4.2)	103	11.1 (8.5–13.8)	8.4 (5.3, 11.4)	<0.001
Poverty ratio category						
<100	104	5.9 (4.7–7.1)	126	10.2 (8.5–11.9)	4.3 (2.2, 6.4)	<0.001
100–199	108	5.5 (4.3–6.7)	141	9.0 (7.1–10.8)	3.5 (1.3, 5.7)	0.002
200–399	101	4.2 (3.4–5.0)	175	7.5 (6.2–8.8)	3.3 (1.7, 4.8)	<0.001
400+	117	3.7 (2.9–4.4)	263	7.6 (6.1–9.1)	4.0 (2.3, 5.7)	<0.001
Physical activity						
Insufficiently active	20	4.3 (2.1–6.4)	52	9.3 (6.2–12.4)	5.0 (1.3, 8.8)	0.009
Sufficiently active	52	5.3 (3.7–6.9)	93	9.1 (6.9–11.4)	3.9 (1.1, 6.6)	0.006
Highly active	140	5.4 (4.3–6.4)	266	7.5 (6.3–8.7)	2.1 (0.5, 3.7)	0.011

a GDM prevalence was calculated with age-standardized to the U.S. population in the year 2000.

We also analyzed the GDM prevalence according to the major risk factors. We found that the prevalence was higher in obese women than in those who were overweight in 2006 (P <0.001), whereas in 2016, the prevalence did not differ significantly (P = 0.23). From 2006 to 2016, the GDM prevalence increased by 4.1% (95% CI, 5.0 to 9.1%) in overweight women, and the corresponding increase tended to be less evident in obese women and women with BMI <25 kg/m^2^, with a change from 7.7 to 10.3% (increased by 2.6%) and 2.8 to 5.7% (increased by 2-9%), respectively (**Table 2** and **Figure 2**).

For the changes in GDM prevalence from 2006 to 2016, women with income below poverty threshold <100% tended to have more increase (4.3%) than those within other income categories including 100–190% (3.5%), 200–399% (3.3%), and >400% (4.0%) (**Table 2** and **Figure 2**).

The GDM prevalence also showed different secular trends from 2006 to 2016 according to physical activity levels. The increase in GDM prevalence appeared more pronounced among women who had insufficient physical activity (5.0%) than among those who had sufficient physical activity (2.1%) (**Table 2** and **Figure 1**).

The prevalence calculated from 11,071 women in 2017 was 8.4 (7.6–9.2) per 100 people, and there was no significant change in the overall and subgroup prevalence compared to that in 2016 (**Table 3**).

**Table 3:** Difference of prevalence in diagnosed Gestational diabetes mellitus of participants in 2016 and 2017.

	2016		2017		*P_difference_*
	Case No.	% (95% CI)	Case No.	% (95% CI)	
All	974	8.2 (7.5–8.9)^a^	799	8.4 (7.6–9.2)	0.99
BMI, kg/m^2^					
<25	241	5.7 (4.8–6.7)	188	5.6 (4.5–6.7)	0.97
25–30	298	9.1 (7.7–10.5)	238	8.6 (7.3–9.9)	0.98
>30	385	10.3 (8.9–11.7)	337	11.7 (9.7–13.6)	0.96
Race/ethnicity					
Hispanic	160	9.5 (7.4–11.6)	135	8.7 (6.6–10.9)	0.96
Non-Hispanic white	583	7.6 (6.7–8.5)	501	8.4 (7.4–9.4)	0.96
Non-Hispanic black	128	7.3 (5.8–8.8)	103	7.0 (5.4–8.6)	0.97
Other race/ethnicity	103	11.1 (8.5–13.8)	60	9.6 (6.8–12.4)	0.93
Poverty ratio category					
<100	126	10.2 (8.5–11.9)	160	10.8 (8.7–12.9)	0.99
100–199	141	9.0 (7.1–10.8)	151	8.4 (6.7–10.2)	0.98
200–399	175	7.5 (6.2–8.8)	235	8.8 (7.4–10.2)	0.93
400+	263	7.6 (6.1–9.1)	225	6.8 (5.0–8.5)	0.93
Physical activity					
Insufficiently active	52	9.3 (6.2–12.4)	218	7.2 (6.0–8.4)	0.99
Sufficiently active	93	9.1 (6.9–11.4)	183	8.6 (7.0–10.2)	0.99
Highly active	266	7.5 (6.3–8.7)	378	8.6 (7.3–9.8)	0.94

a GDM prevalence was calculated with age-standardized to the U.S. population in the year 2000.

## Discussion

In this study of the nationally representative data of U.S. populations, we found that the prevalence of GDM increased from 4.6% in 2006 to 8.2% in 2016, with a relative increase rate of 78%. Our stratified analysis revealed that the increase tended to be more pronounced among women who were non-white, overweight, had insufficient activity, and had lower socioeconomic status. The prevalence of GDM has reached a steady rate in 2017 since 2016.

Several previous studies have reported an increasing trend of GDM prevalence in the United States between 1979 and 2010 (5, 10, 13, 17). In a national survey among hospitalized women, the prevalence of GDM increased from 1.9% in 1989–1990 to 4.2% in 2003–2004 (11). Another regional study, the Kaiser Permanente of Colorado (KPCO) study, showed a similar trend, with the prevalence of GDM increasing from 2.1% in 1994 to 4.1% in 2002 (13). An increasing trend of GDM prevalence was also observed in other studies (17, 18), while inconsistent observations were also reported. For example, in PRAMS, no significant change was observed between 2007–2008 (8.1%) and 2009–2010 (8.5%) (5). Compared with previous national studies, in which the GDM prevalence increased from 1979 to 2010 with an absolute increase of ~1.8% per decade (10, 11), our data indicated that the GDM prevalence continuously increased from 2006 to 2016, and the absolute increase rate (3.6% per 10 years) appeared to be accelerated as compared with previous years.

The significant increase in the prevalence of GDM in the past 10 years might be attributed to the concurrent changes in multiple risk factors, such as increased prevalence of overweight and obesity (19, 20), advanced maternal age (21) and the growth of minority populations that had a higher risk of GDM (22). Overweight and obesity are major risk factors for developing GDM (23). Between 2006 and 2016, the increased prevalence of overweight and obesity persisted among adult women in the United States (20, 24), and overweight and obesity were considered to be the major driving forces for the increase in GDM prevalence (12, 25). Intriguingly, we found that the increase in GDM prevalence was more pronounced among overweight rather than obese women. We assumed that changes in certain risk factors might more likely increase GDM risk among overweight women than obese women. For example, several studies showed that the associations of gestational weight gain (GWG) with gestational impaired glucose tolerance and GDM were stronger among overweight women than obese women (26, 27). Therefore, the greater increase in GDM prevalence among overweight women than obese women might be partly due to the increasing excessive GWG over the past decades (28, 29). Even though the increasing prevalence of GDM was observed in all racial/ethnic groups, we noted that non-Hispanic whites showed the least increase, while the increase was most evident among women of other race/ethnicity (more than 72% were Asian). Given the growth of minority populations in the past decade, both observed in the present study and reported previously (22), our data suggest the changing racial/ethnic profiles might partly explain the increase in GDM prevalence. Additionally, we found that women who had insufficient physical activity tended to a greater increase in GDM. Our results are in agreement with findings from several prospective studies in which regular physical activity before pregnancy was related to a reduced GDM risk (30–32). Moreover, we found that women with low socioeconomic status had a greater increase in GDM prevalence than those with high socioeconomic status. Socioeconomic status has been inversely correlated with the risk of GDM. It was reported that the risk of GDM among women living in the lowest socioeconomic regions was approximately two-thirds higher than that of women living in the highest socioeconomic regions (33). Women with low socioeconomic status have limited access to effective medical care (34), and low socioeconomic status could be considered as a marker for other established risk factors for GDM, such as obesity (35).

Changed diagnostic criteria or screening strategies might also partly account for the observed increase in GDM prevalence (1). The GDM diagnosis in 2006 was based on the World Health Organization diagnostic criteria with 1-h 50 H GCT plus 3-h 100 g OGTT (two-step) (36). After 2011, the year when the International Association of Diabetes and Pregnancy Study Groups (IADPSG) criteria (with only one step: 2-h 75G OGTT) (1) for screening and diagnosis of GDM were recommended by ADA guidelines, leading to an increase in the prevalence based on change criteria in some areas (37–39). Since a previous study also showed the GDM prevalence increased from 1998 to 2010 using consistent diagnostic criteria (10); and not all women in 2016 had their first pregnant after 2011, this change in diagnostic criteria alone might not explain the observed increase in GDM prevalence.

We found no change in the prevalence of GDM between 2016 and 2017. The potential slowing of the increase in GDM prevalence may relate to the slowing of BMI (40), which is a major risk factor for GDM. Overall, the prevalence of obesity increased from 35.7% in 2005–2006 to 40.5% in 2013–2014 among women (41). But the increase might slow down in certain years. For example, there was no significant change in obesity prevalence between 2009–2010 and 2011–2012 (42). Another explanation may be policy and advocacy for healthier lifestyles that could attenuate the adverse effects of other GDM-related risk factors may be another explanation. The growing number of noncommunicable diseases (NCDs) and related risk factors might also impact the GDM prevalence (43). Recognizing the burden of NCDs, the WHO Global NCD Action Plan 2013–2020 has been developed to prevent and control NCDs and their risk factors and determinants, which might to a certain extent decrease the prevalence of GDM. Additionally, the relatively short period between 2016 and 2017 may also account for the non-significant change.

In the short and long term, GDM has been linked with a wide range of adverse health consequences for women and their offspring. For example, GDM has been related to a higher risk of type 2 diabetes and cardiovascular disease in women. Additionally, offspring of mothers with GDM are prone to various adverse outcomes such as macrosomia, hypoglycemia, and type 2 diabetes later in life (44). Our study identified the subgroups at high GDM risk, namely, women of the minority (e.g., Hispanic women) or those who are overweight, have insufficient activity, and low family income; and these findings call for more attention and intervention by healthcare workers to prevent the development of GDM or its adverse outcomes in these high-risk women.

### Strengths and Limitations of the Study

As far as we are aware, this study is the first to report the nationally representative data of GDM prevalence and trends in the past decade in the United States. A major strength lies in the ability of our comprehensive analysis to display the trend of GDM prevalence by race/ethnicity and other demographic and socioeconomic characteristics. Our study has several limitations. A major limitation is that self-reported physician-diagnosed GDM may under or overestimate the true prevalence of diagnosed GDM. However, the sensitivity and specificity of self-reported GDM have been reported in previous studies (16), and the self-reported GDM in NHIS has been widely used in other studies (45, 46). Another potential limitation of this study is that information was limited on which criteria were used for the diagnosis of GDM. Thus, we could not determine to what extent the changed criteria might account for the observed increase. Additionally, a new sample design was implemented for the 2016 and 2017 NHIS and sample areas were reselected to consider changes in the distribution of the U.S. population since 2006. This might also affect the estimate of the GDM prevalence. Moreover, data on institutionalized people, for whom the GDM prevalence might differ from those in the general population, was not available in the NHIS. Lastly, the relatively small sample size of a subgroup decreased the power to test the differences among the changes in subgroups.

## Conclusions

Our study provides evidence that the prevalence of GDM has continuously increased among U.S. women in the past decade, and the increase tended to be more marked among the minority populations and those who were overweight, had insufficient activity, and had an income below the poverty threshold.

## Data Availability Statement

Publicly available datasets were analyzed in this study. This data can be found here: https://www.cdc.gov/nchs/nhis/index.htm.

## Author Contributions

LQ conceptualized and designed the study, critically reviewed the manuscript, and approved the final submission. TZ conceptualized and designed the study, contributed to data cleaning and the statistical analysis, and drafted the initial manuscript. SD, DS, YH, GH, LS, XP, and XS contributed to data cleaning and the statistical analysis, reviewed and revised the manuscript, and approved the final manuscript as submitted. All authors listed have made a substantial, direct, and intellectual contribution to the work and approved it for publication.

## Funding

The study was supported by grants from the National Heart, Lung, and Blood Institute (HL071981, HL034594, and HL126024), the National Institute of Diabetes and Digestive and Kidney Diseases (DK091718, DK100383, DK115679, and DK078616), the Fogarty International Center (TW010790), the Boston Obesity Nutrition Research Center (DK46200), the United States–Israel Binational Science Foundation Grant 2011036, the National Natural Science Foundation of China (No. 31971147), the Guangdong Basic and Applied Basic Research Foundation (No. 2021B1515020047), and the Shenzhen Science and Technology Innovation Commission (JCYJ20200109142446804). LQ was a recipient of the American Heart Association Scientist Development Award (0730094N). All investigators are independent from funders.

## Conflict of Interest

The authors declare that the research was conducted in the absence of any commercial or financial relationships that could be construed as a potential conflict of interest.

## Publisher’s Note

All claims expressed in this article are solely those of the authors and do not necessarily represent those of their affiliated organizations, or those of the publisher, the editors and the reviewers. Any product that may be evaluated in this article, or claim that may be made by its manufacturer, is not guaranteed or endorsed by the publisher.
